# SIRT2 Deficiency Modulates Macrophage Polarization and Susceptibility to Experimental Colitis

**DOI:** 10.1371/journal.pone.0103573

**Published:** 2014-07-29

**Authors:** Giuseppe Lo Sasso, Keir Joe Menzies, Adrienne Mottis, Alessandra Piersigilli, Alessia Perino, Hiroyasu Yamamoto, Kristina Schoonjans, Johan Auwerx

**Affiliations:** 1 Laboratory for Integrative and Systems Physiology, École Polytechnique Fédérale de Lausanne, Lausanne, Switzerland; 2 Institute of Animal Pathology, University of Bern, Bern, Switzerland; 3 School of Life Sciences, École Polytechnique Fédérale Lausanne, Lausanne, Switzerland; 4 Department of Biomedical Informatics Division of Health Sciences, Osaka University Graduate School of Medicine, Suita-City, Osaka, Japan; Charité, Campus Benjamin Franklin, Germany

## Abstract

**Background:**

SIRT2 belongs to a highly conserved family of NAD^+^-dependent deacylases, consisting of seven members (SIRT1–SIRT7), which vary in subcellular localizations and have substrates ranging from histones to transcription factors and enzymes. Recently SIRT2 was revealed to play an important role in inflammation, directly binding, deacetylating, and inhibiting the p65 subunit of NF-κB.

**Methods:**

A *Sirt2* deficient mouse line (*Sirt2^−/−^*) was generated by deleting exons 5–7, encoding part of the SIRT2 deacetylase domain, by homologous recombination. Age- and sex-matched *Sirt2^−/−^* and *Sirt2^+/+^* littermate mice were subjected to dextran sulfate sodium (DSS)-induced colitis and analyzed for colitis susceptibility.

**Results:**

*Sirt2^−/−^* mice displayed more severe clinical and histological manifestations after DSS colitis compared to wild type littermates. Notably, under basal condition, *Sirt2* deficiency does not affect the basal phenotype and intestinal morphology *Sirt2* deficiency, however, affects macrophage polarization, creating a pro-inflammatory milieu in the immune cells compartment.

**Conclusion:**

These data confirm a protective role for SIRT2 against the development of inflammatory processes, pointing out a potential role for this sirtuin as a suppressor of colitis. In fact, SIRT2 deletion promotes inflammatory responses by increasing NF-κB acetylation and by reducing the M2-associated anti-inflammatory pathway. Finally, we speculate that the activation of SIRT2 may be a potential approach for the treatment of inflammatory bowel disease.

## Introduction

Intestinal bowel disease (IBD) is a chronically recurring inflammatory disorder arising from genetic predispositions and/or environmental or immunological modifying factors [1], [2] that negatively affect the interaction between the commensal microflora and the intestinal mucosa [3]. The two most common forms of IBD are Crohn's disease (CD) and ulcerative colitis (UC). These diseases often result in morbidity due to a high incidence of diarrhea, abdominal pain, rectal bleeding and malnutrition [1]. Despite significant progresses, our understanding of the inflammatory regulators that contribute to the pathogenesis of IBD is still limited.

Recently, SIRT2, an NAD^+^-dependent sirtuin deacetylase, was revealed to play an important role in inflammation [4], [5], [6], [7]. SIRT2 belongs to a highly conserved family of NAD^+^-dependent enzymes, consisting of seven members (SIRT1–SIRT7), which vary in subcellular localizations and have substrates ranging from histones to transcription factors and enzymes [8], [9]. SIRT2 is primarily a cytosolic protein, but can shuttle into the nucleus [10], [11], thus explaining its ability to deacetylate both cytosolic (e.g. α-tubulin) [11] and nuclear (e.g. histones) [10] substrates. In the context of inflammation, SIRT2 was shown to directly bind and deacetylate the p65 subunit of NF-κB [4], a major transcriptional regulator of the inflammatory response [12]. Accordingly, p65 is hyperacetylated in *Sirt2^−/−^* mouse embryonic fibroblasts following TNFα stimulation, resulting in NF-κB-dependent gene activation and increased apoptosis [4]. Furthermore, *in vivo* experiments show that SIRT2 is an important inhibitor of microglia-mediated inflammation in the brain [5], and of inflammatory factors leading to arthritis [6]. These discoveries led to the use of SIRT2 as an anti-inflammatory therapeutic target, as was recently demonstrated by using a permeative protein, Pep-1, to transduce SIRT2 into epithelial cells [7]. Transduction of cells with Pep-1-SIRT2 reduced inflammation by attenuating the expression of cytokines and activation of both NF-κB and mitogen activated protein kinases (MAPKs). These recent findings prompted us to examine the potential contribution of SIRT2 in the development of IBD.

In the present study, we demonstrate that SIRT2 is critical for modulating macrophage polarization and intestinal permeability, thereby inhibiting the development of colitis. More specifically, SIRT2 knockout (*Sirt2^−/−^*) mice developed more severe colitis when exposed to the chemical colitis inducer, dextran sulfate sodium (DSS) [13]. This phenotype appears to be consequent to a hyper-activated immune cell compartment with secondary changes in the intestinal epithelium. In fact, the intestinal histological appearance and the expression of genes involved in intestinal permeability are similar between untreated *Sirt2^−/−^* and wild type (*Sirt2^+/+^*) mice. However, *Sirt2^−/−^* bone marrow-derived macrophages (BMDMs) show an activation of inflammatory genes, along with the hyperacetylation of the NF-κB subunit p65, confirming a pro-inflammatory state in untreated mice. Therefore, since sirtuins are considered druggable enzymes, our results suggest that targeting SIRT2 may be of particular interest for the management of IBD.

## Materials and Methods

### Generation of mice

The generation of *Sirt2* floxed (*Sirt2^L2/L2^*) mice has been described before [14]. *Sirt2^L2/+^* mice (heterozygote conditional animals that have the conditional allele with Lox sites) were selected and intercrossed with CMV-Cre mice to delete the *Sirt2* gene in the male germline. Offspring with a deleted allele (*Sirt2^L−/+^* mice) were then mated to C57BL/6J mice in order to remove the Cre-transgene. The resulting *Sirt2^L−/+^* offspring without the Cre transgene were then backcrossed for 10 generations onto commercial C57BL/6J mice purchased from the Jackson Laboratory to generate heterozygous *Sirt2^L−/+^* mice, from now on simply termed *Sirt2^−/+^* mice. Breedings were only performed with such congenic heterozygous *Sirt2^−/+^* mice to generate the cohorts of male *Sirt2^−/−^* and *Sirt2^+/+^* littermates used for the *in vivo* studies. Animal experiments were done in accordance with institutional and Swiss guidelines and approved by the authorities of the Canton of Vaud. Moreover, all animal experiments were conformed to the Swiss Animal Welfare legislation and reviewed by the State Ethical Board of the Canton de Vaud (Animal Welfare Act 2005; Project License N° 2463.1 licensed to Prof. Johan Auwerx). Mice were euthanatized using a brief exposure to CO_2_. This method leads to quick and painless asphyxiation of mice. All the experiments were carried out from January 2013 to May 2014.

### Antibodies

FACS analysis of mesenteric lymph node cells: CD4-APC (eBioscience, clone GK1.5), TCRb-PE (eBioscience, clone H57-597), CD69-biotin (eBioscience, clone H1.2F3), Streptavidin-FITC, (eBioscience). Immunohystochemistry: F4/80 (AbD Serotec; MCA497). Western blot: SIRT2 (H-95, SantaCruz sc-20966), Acetyl-NF-κB (Acetyl-K310, Abcam, ab19870), phospho-IKbα (ser32/36, Cell Signaling 9246), IKbα (L35A5, Cell Signaling 4814), Hsp90 (BD Transduction Laboratories, 610418).

### DSS-induced colitis

DSS-induced colitis was induced as previously described [15] using 2.5% dextran sulfate sodium (36–50 KDa, MP Biomedicals) solution in water. Daily changes in body weight were assessed. Rectal bleeding was scored on a scale from 0 to 5, indicating no (0) or highly severe (5) rectal bleeding. Colons were snap-frozen or fixed with 4% Forma-Fixx (Thermo scientific) and embedded in paraffin. *In vivo* intestinal permeability was examined in mice as was previously described [16].

### Mesenteric lymph nodes isolation and FACS analysis

Mesenteric lymph nodes (MLNs) were dissected from DSS-treated mice and a single cell suspension was obtained by passing the MLNs through a 40 µm filter. After counting, the cell suspension was incubated in HBSS containing 25 mM HEPES and the primary antibody. Incubation with anti-biotin antibody was performed when indicated. FACS analysis was performed on CyAn ADPS analyzers (Beckman Coulter).

### Cytokine measurement

Blood was collected from mice at sacrifice and plasma EDTA was obtained after centrifugation at 3000 rpm for 10 minutes at 4°C. Cytokine concentration in the plasma was measured by using the Mouse Proinflammatory Panel 1 kit (Meso Scale Diagnostics) following manufacturer's instructions.

### mRNA extraction and RT-qPCR analysis

RNA was isolated from colon or bone marrow-derived macrophages using the TriPure reagent (Roche) according with manufacturer's instructions. cDNA was generated from 1 µg of total RNA using QuantiTect Reverse Transcription Kit (Qiagen). qRT-PCR was carried out using LightCycler 480 SYBR Green I Master Mix (Roche) and analyzed through ΔΔCT calculation. Values were normalized to Cyclophilin expression. Primers: Il6 (Fw GAGGATACCACTCCCAACAGACC; Rv AAGTGCATCATCGTTGTTCATACA); Il1β (Fw CAACCAACAAGTGATATTCTCCATG; Rv GATCCACACTCTCCAGCTGCA); Tnfα (Fw GGGACAGTGACCTGGACTGT; Rv AGGCTGTGCATTGCACCTCA); Occludin (Fw AGCCTCGGTACAGCAGCAAT; Rv CCTTCGTGGGAGCCCTTT); Claudin-1 (Fw CATAGGCACGGACTTCTGGTA; Rv CCAGGCGATTTTATTCGAGTCAC); Zo1 (Fw GACTCCAGACAACATCCCGAA; Rv AACGCTGGAAATAACCTCGTTC); Jam-A (Fw ACCCTCCCTCCTTTCCTTAC; Rv CTAGGACTCTTGCCCAATCC); Mcp-1 (Fw AGGTCCCTGTCATGCTTCTG; Rv GCTGCTGGTGATCCTCTTGT); Gata3 (Fw CTCGGCCATTCGTACATGGAA; Rv GGATACCTCTGCACCGTAGC); Arg1 (Fw GCAGAGGTCCAGAAGAATGGAA; Rv GCGTGGCCAGAGATGCTT); CD11c (Fw ACGTCAGTACAAGGAGATGTTGGA; Rv ATCCTATTGCAGAATGCTTCTTTACC); Il4R (Fw TCTGCATCCCGTTGTTTTGC; Rv GCACCTGTGCATCCTGAATG); Il10 (Fw CATGGCCCAGAAATCAAGGA; Rv GGAGAAATCGATGACAGCGC), Cyclophilin (Fw CAGGGGAGATGGCACAGGAG; Rv CGGCTGTCTGTCTTGGTGCTCTCC).

### Intestine isolation, immunohistochemistry, and scoring

Colon was excised and collected from the cadavers, carefully slit opened longitudinally along the antimesenteric side. Feces were removed from the lumen and each segment was rolled on a wooden stick with the serosal side adhering to it. The Swiss rolls were then placed in 4% Forma-Fixx for 24 hours. Paraffin embedding was then performed. 5 µm thick sections were cut from paraffin blocks. Slides were respectively stained with hematoxylin/eosin for morphologic analysis of the tissue and with F4/80 for quantification of macrophages. The number of F4/80 positive cells in the tunica mucosa was scored as count of number of positive cells with clear morphology in 6 random fields at 400× magnification. Areas presenting artifacts were excluded from the analysis. The histopathological evaluation of the specimens was performed by a European board certified veterinary pathologist in a blinded fashion according with the following scoring: Erosion/ulceration: 5–10% (1); 20–30% (2); 40–50% (3); 60–70 (4); 80–90 (5); 100% (6). Inflammation severity: minimal (1); mild (2); moderate (3); severe (4); very severe (5). Mural involvement: mucosa (1); submucosa (2); tunica muscularis (3); serosa (transmural) (4); transmural reaching mesentery (steatitis) (5). Damage distribution: <25% (1); 26–50% (2); 51–75% (3); >75% (4).

### Bone marrow-derived macrophages (BMDM) isolation and stimulation

Bone marrow derived macrophages (BMDMs) were isolated from femurs and tibias of sibling 8- to 10-week-old male *Sirt2^+/+^* and *Sirt2^−/−^* sibling mice. Cells were plated on bacteriological plastic plates in macrophage growth medium consisting of RPMI-1640 (Invitrogen), 1 mM sodium pyruvate (Invitrogen), 1× non essential amino acids (Invitrogen), 5 mM penicillin/streptomycin (Invitrogen), 10% heat-inactivated foetal bovine serum (GE Healthcare) supplemented with 10% L-cell-conditioned medium as a source of CSF-1. After one day, non-adherent cells were collected and seeded at 10^5^ cells/ml in bacteriological plates and grown for 7 days. Differentiated BMDMs were stimulated with LPS (10 ng/ml) or with IL-4 (10 nM) for 6 and 24 h, respectively.

### Statistical Analyses

The comparison of different groups was carried out using a Student's *t*-test and a two-way analysis of variance (ANOVA), and differences with a *P*<0.05 were considered statistically significant (*P<0.05, ** *P*<0.01, *** *P*<0.001).

## Results

### *Sirt2^−/−^* mice have normal colon morphology

**Figure 1:**
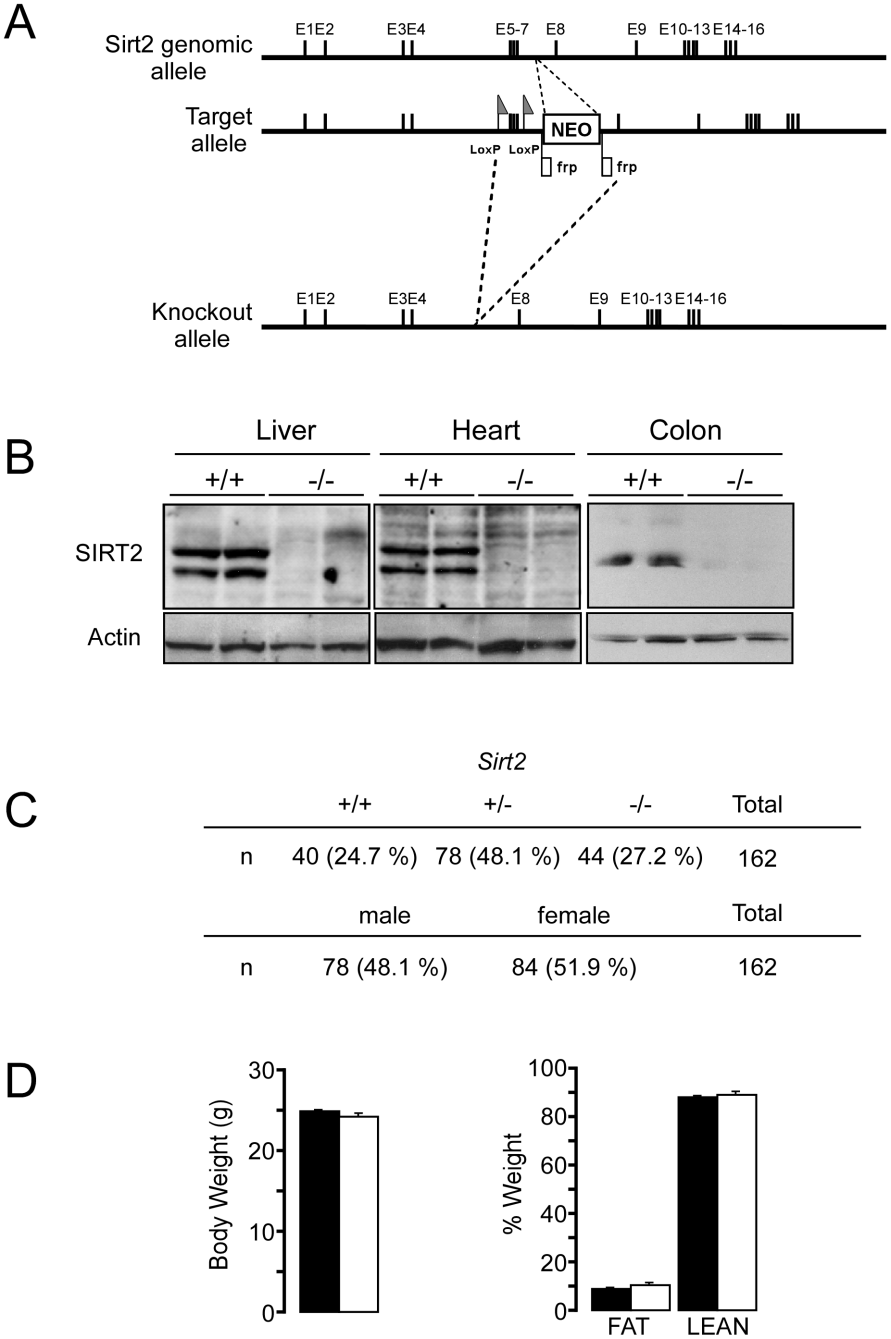
Generation and characterization of the *Sirt2^−/−^* mouse model. (A) Schematic representation of the gene targeting strategy for exons 5–7 of the *Sirt2* gene. (B) Western blot analysis of SIRT2 expression in the liver, heart, and colon of *Sirt2^+/+^* and *Sirt2^−/−^* mice. (C) Genotype and sex distributions of newborn mice summarized from 162 colonies. (D) Body weight and body composition of *Sirt2^+/+^* and *Sirt2^−/−^* mice.

**Figure 2:**
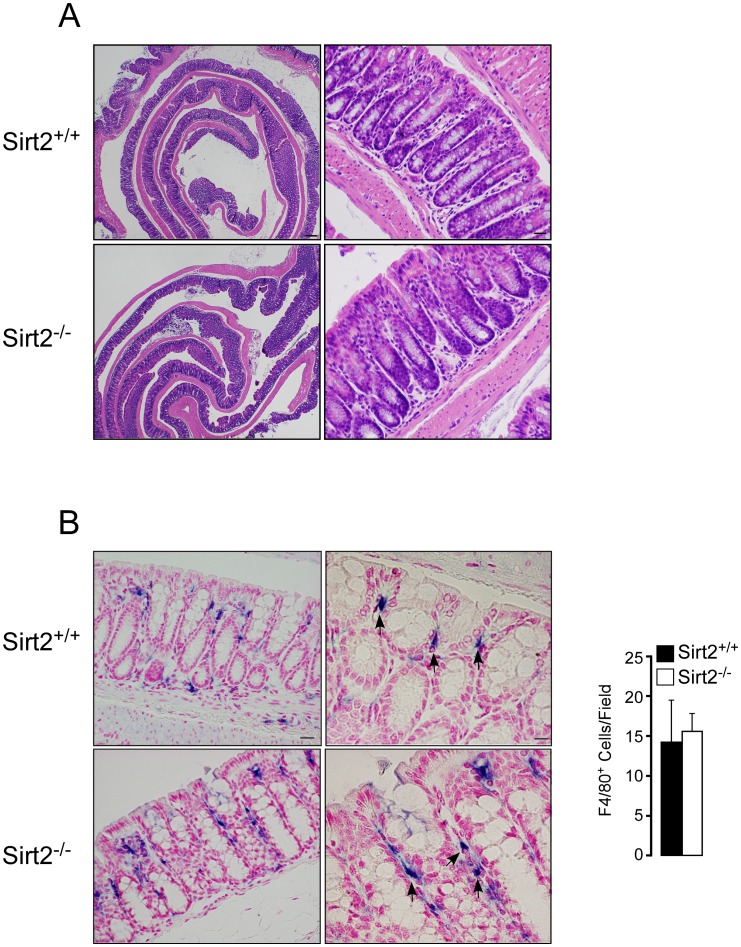
*Sirt2^−/−^* and *Sirt2^+/+^* mice colons are morphologically similar under normal conditions. (A and B) Representative images of hematoxylin/eosin (A) and F4/80 staining (B) of the colon in *Sirt2^+/+^* and *Sirt2^−/−^* mice. Number of F4/80^+^ cells are shown (right panel of B). n = 3/group. Scale bar = 20 µm.

To study the possible involvement of SIRT2 in the pathogenesis of colitis, we first generated a *Sirt2* deficient mouse line (*Sirt2^−/−^*) by targeting part of the SIRT2 deacetylase domain (exons 5–7), by homologous recombination in ES cells (Figure 1A). *In silico* translation of this *Sirt2*-deletion product, results in short incomplete peptides when examining all potential reading frames. As a result, no SIRT2 protein was detected in any of the tissues analyzed from *Sirt2^−/−^* mice (Figure 1B). The offspring of heterozygous *Sirt2^+/−^* breeders were born under normal Mendelian (+/+ : +/− : −/− = 24.7% : 48.1% : 27.2%) and sex ratios (male : female = 48.1% : 51.9%) (Figure 1C), with no differences observed in body weight or body composition between *Sirt2^−/−^* and *Sirt2^+/+^* mice (Figure 1D). Colon morphology of *Sirt2^+/+^* and *Sirt2^−/−^* mice was then examined under basal conditions. The morphological analysis of the colons did not reveal any qualitative and/or quantitative differences, in terms of crypts depth, epithelial cell differentiation, wall thickness or density of leukocytes in the colon between the two genotypes (Figure 2A). Moreover, the immunohistochemical quantification of macrophages (F4/80^+^ cells) within the tunica mucosa of the colon revealed no differences between the two groups (Figure 2B). Thus, *Sirt2* deficiency does not appear to affect the basal phenotype and intestinal morphology of *Sirt2^−/−^* mice when compared with their wild type counterparts.

### *Sirt2* deficiency increases the severity of DSS-induced colitis

**Figure 3:**
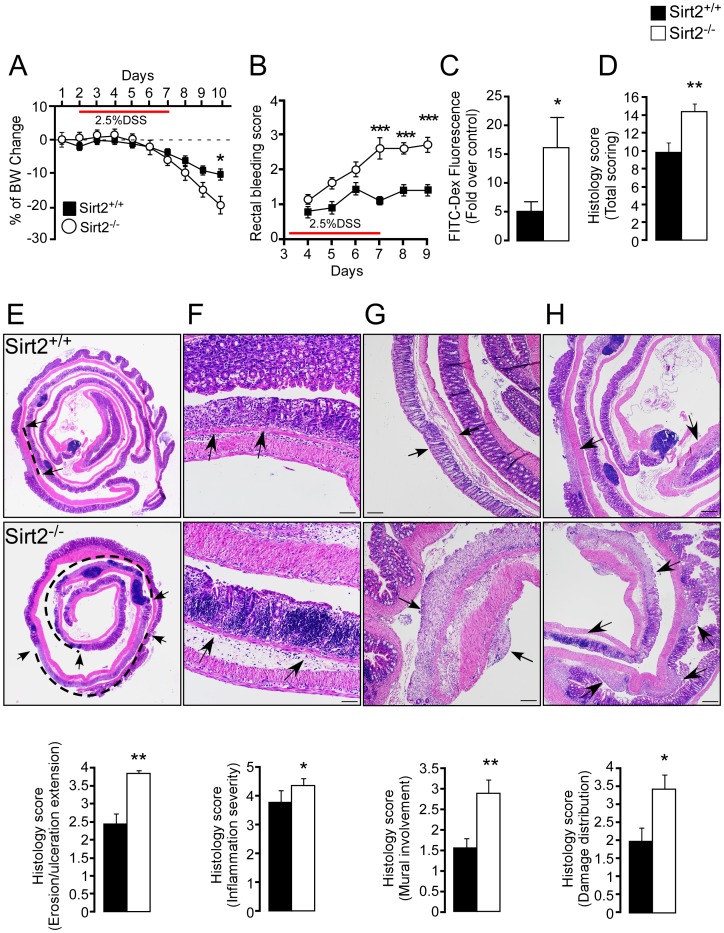
*Sirt2^−/−^* mice are more sensitive to DSS-induced colitis compared to *Sirt2^+/+^* animals. (A–D) Severity of DSS-induced colitis was determined by body weight change (A), rectal bleeding scores (B), intestinal permeability (C), and histological scores (D) in *Sirt2^+/+^* and *Sirt2^−/−^* mice. n = 10/group. (E–H) Histological changes in the intestine of the DSS-treated *Sirt2^+/+^* and *Sirt2^−/−^* mice. Representative images demonstrating the extension of erosion/ulceration (arrows and dashed line indicate the extent of ulceration) (E), inflammation severity (arrows indicate leukocytic infiltrate and follicular aggregates) (F), mural involvement (arrows indicate transmural infiltration) (G), and damage distribution (arrows indicate sites of damage) (H). Corresponding histological scores are shown (lower panels). n = 10/group. Scale bar = 20 µm. Results are expressed as the mean ± SEM. *P<0.05; **P<0.01; ***P<0.001.

To test whether *Sirt2* deficiency contributes to the development of colitis, we exposed *Sirt2^+/+^* and *Sirt2^−/−^* mice to DSS to chemically induce intestinal inflammation [17]. *Sirt2*-deficiency significantly accelerated body weight loss (Figure 3A) and increased the rectal bleeding score (Figure 3B) after DSS treatment. Furthermore, plasma levels of FITC-conjugated dextran were markedly increased in *Sirt2^−/−^* mice (Figure 3C), indicating increased intestinal epithelial permeability. The total histological score, accounting for the overall severity of intestinal inflammation, was significantly higher in the colons of *Sirt2^−/−^* mice (Figure 3D). In particular, *Sirt2^−/−^* mice showed a three-fold increase in the extent of epithelial cell loss (ranging from erosion to ulceration) compared to *Sirt2^+/+^* littermates (Figure 3E). The inflammation severity, evaluated by the leukocytic infiltration density, was also significantly increased in *Sirt2^−/−^* mice, with a strong tendency to form follicular aggregates within the mucosal layer (Figure 3F). Finally, more frequent transmural infiltration of inflammatory cells was observed in the tunica serosa (peritonitis) and even in the mesenteric fat tissue (steatitis) of *Sirt2^−/−^* mice (Figure 3G), while mural extension occurred less in *Sirt2^+/+^* mice. The overall distribution of intestinal damage was often multifocal for both groups, although, in *Sirt2^−/−^* mice the percentage of affected tissue was significantly higher (Figure 3H). Thus, DSS resulted in a heavily exacerbated form of colitis in the *Sirt2^−/−^* mice when compared to the *Sirt2* ^+/+^ animals.

**Figure 4:**
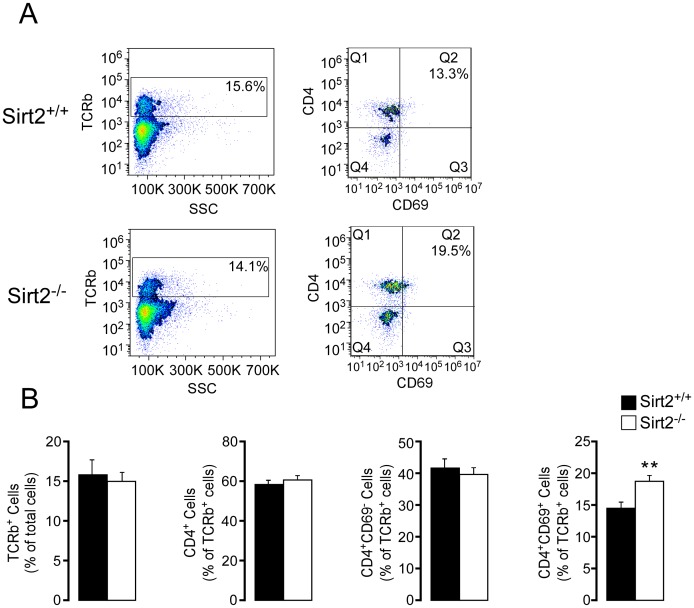
CD4^+^CD69^+^ T cells are increased in mesenteric lymph nodes from *Sirt2^−/−^* mice with DSS-induced colitis. (A) Representative images of FACS analysis demonstrating TCRb^+^ cells (left), and their composition sorted by CD4 and CD69 staining (right). (B) The composition of MLNs is compared between *Sirt2^+/+^* and *Sirt2^−/−^* mice; TCRb^+^, CD4^+^, CD4^+^CD69^−^, and CD4^+^CD69^+^ cells. n = 10/group. Results are expressed as the mean ± SEM. *P<0.05; **P<0.01; ***P<0.001.

Furthermore, the cell composition of mesenteric lymph nodes, as determined by FACS analysis, was distinct between the two genotypes. Although no difference was observed in the total amount of CD4^+^ T lymphocytes, *Sirt2^−/−^* mice had an increased proportion of activated lymphocytes (CD4^+^/CD69^+^) compared to the control mice (Figure 4A–B), consistent with an enhanced inflammatory response.

Taken together these data show that SIRT2 plays an important role in protecting from DSS-induced colitis and that its deficiency exacerbates the clinical and pathological severity of disease progression.

### *Sirt2* deficient mice show increased levels of pro-inflammatory cytokines

**Figure 5:**
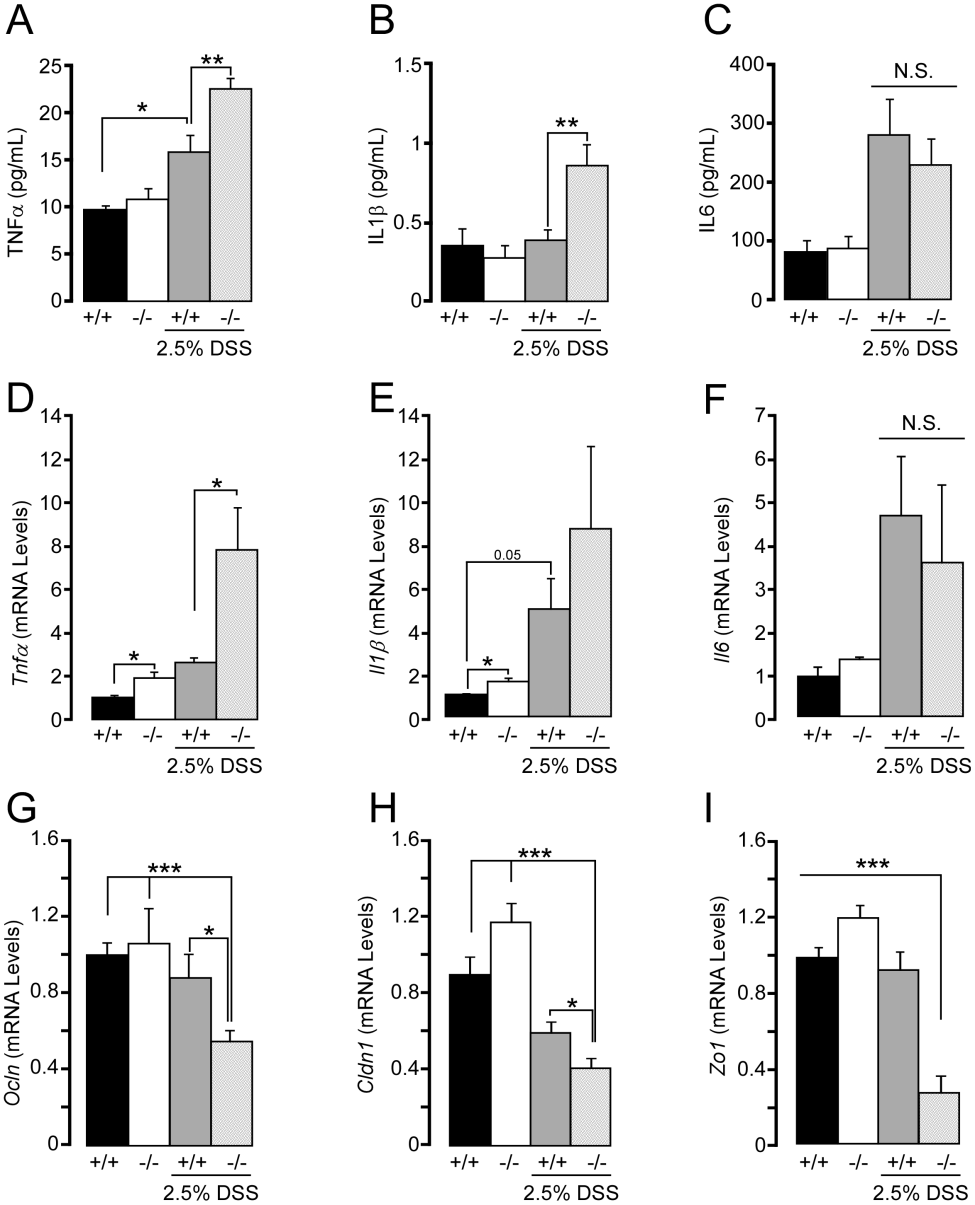
Plasma cytokine levels and cytokine mRNA levels in the colon of *Sirt2^−/−^* mice with DSS-induced colitis. (A–C) Measurements of serum cytokine levels in *Sirt2^+/+^* (+/+) and *Sirt2^−/−^* (−/−) mice after DSS-induced colitis; TNFα (A), IL1β (B), and IL6 (C). (D–E) Cytokine mRNA levels in the colon of *Sirt2^+/+^* and *Sirt2^−/−^* mice before and after DSS-induced colitis. *Tnfα* (D), *Il1*β (E), and *Il6* (F). (G–H) mRNA levels of the genes related to maintenance of intestinal permeability, *Ocln* (G), *Cldn1* (H), *Zo1*(I) in *Sirt2^+/+^* and *Sirt2^−/−^* mice with or without DSS treatment. n = 10/group (with DSS treatment); n = 5/group (without DSS treatment). Results are expressed as the mean ± SEM. *P<0.05; **P<0.01; ***P<0.001.

Pro-inflammatory cytokines such as TNFα, IL1β, and IL6 play pivotal roles in the pathogenesis of colitis [18]. Since colitis is a systemic inflammatory disease, we measured cytokine levels in the plasma of *Sirt2^+/+^* and *Sirt2^−/−^* mice, during basal conditions and after DSS treatment. Under normal conditions both genotypes of mice showed similar levels of plasma cytokines (Figure 5A–C). However, after DSS treatment, plasma levels of TNFα and IL1β were more elevated in *Sirt2^−/−^* mice, compared to the *Sirt2^+/+^* animals (Figure 5A–B), whereas IL6 levels, although increased by DSS treatment, remained indistinguishable between the two genotypes (Figure 5C). Unlike the plasma cytokine levels, the colon mRNA levels of *Tnfα* and *Il1β* were already modestly higher in *Sirt2^−/−^* mice in basal conditions (Figure 5D–E). Following DSS, an induction in the expression of the mRNAs coding for these cytokines occurred in both genotypes; however, only the induction of *Tnfα* transcript was significantly different in *Sirt2^−/−^* from *Sirt2^+/+^* mice (Figure 5D). Notably, *Il6* mRNA levels were again indistinguishable between both genotypes, both before and after DSS (Figure 5F).

Together with inflammatory mediators, tight junctions (TJs), which control epithelial paracellular permeability, have a fundamental role in IBD development and progression. Patients with IBD have both disrupted intestinal epithelial barrier function and altered expression of TJ proteins [19], [20]. Thus, we analyzed the transcript levels of some principal TJ proteins, including *Occludin* (*Ocln*), *Claudin-1* (*Cldn1*) and *Zona Occludens-1* (*Zo1*) [21]. Under basal conditions no differences in the expression of the aforementioned genes were observed between *Sirt2^+/+^* and *Sirt2^−/−^* mice, confirming that *Sirt2* deficiency in colon tissue does not directly affect intestinal physiology (see also Figure 2). However, upon the DSS challenge a significant decrease in the transcripts of these TJ proteins was observed in *Sirt2^−/−^* mice (Figures 5G–H). Interestingly, only *Cldn1* expression decreased in both genotypes following DSS treatment (Figure 5H), yet the percent reduction was significantly higher in *Sirt2^−/−^* mice. These results clearly indicate that *Sirt2* deficiency promotes inflammatory processes and intestinal permeability during the development DSS-dependent colitis.

### *Sirt2* deficiency alters immune cell activation following a DSS-challenge

**Figure 6:**
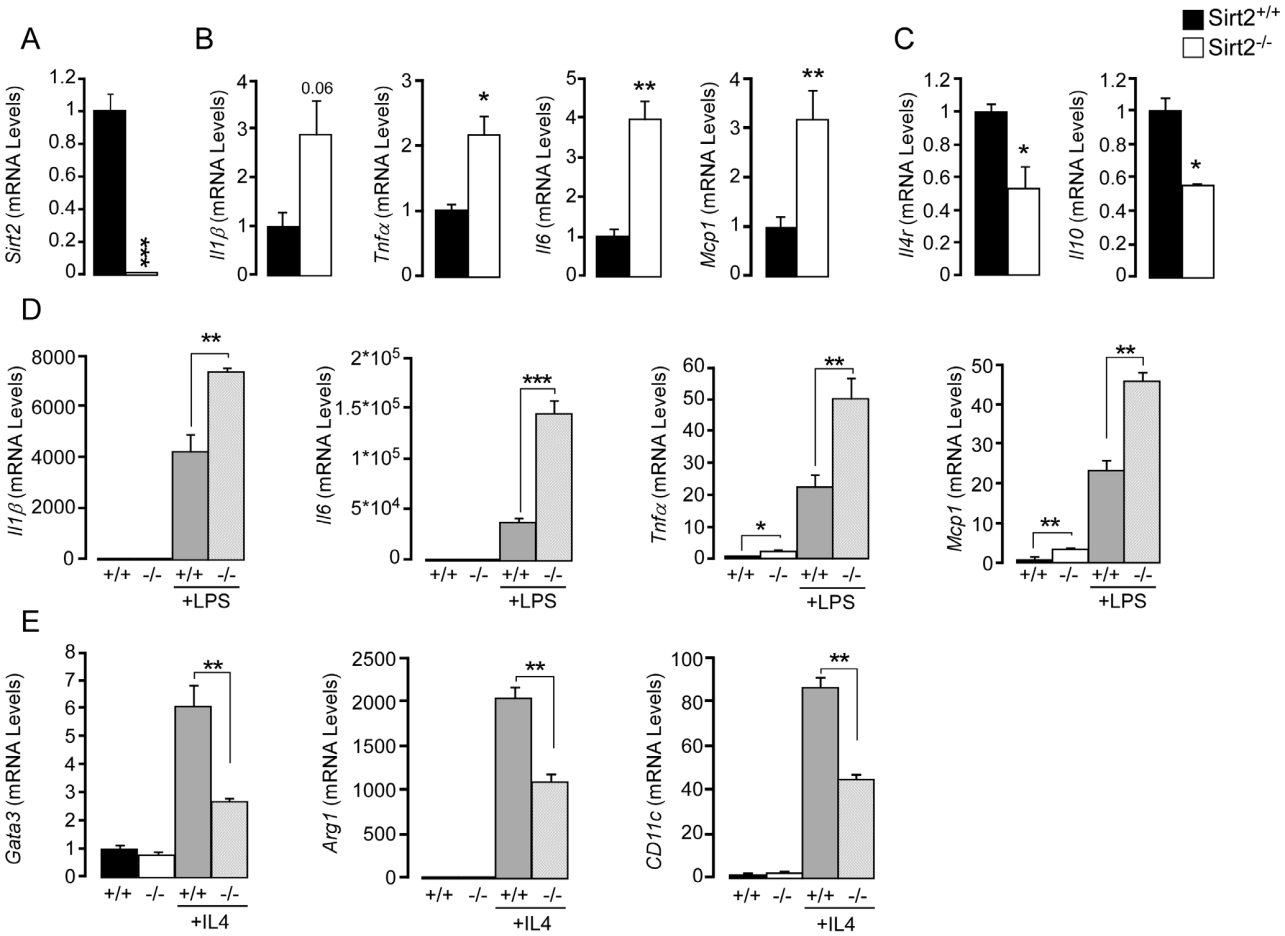
Increased mRNA levels of pro-inflammatory genes and decreased levels of anti-inflammatory genes in *Sirt2^−/−^* mouse-derived BMDMs. (A) *Sirt2* mRNA levels in *Sirt2^+/+^* (+/+) and *Sirt2^−/−^* (−/−) mice. (B and C) mRNA levels of pro-inflammatory cytokine genes (*Il1β*, *Tnfα*, *Il6*, *Mcp1*) (B) and anti-inflammatory genes (*Il4r*, *Il10*) (C) in BMDMs from *Sirt2^+/+^* and *Sirt2^−/−^* mice under basal conditions. n = 3/group. (D) mRNA levels of pro-inflammatory genes in BMDMs from *Sirt2^+/+^* and *Sirt2^−/−^* mice upon LPS treatment. (E) mRNA levels of *Gata3*, *Arg1*, and *Cd11c* in BMDMs from *Sirt2^+/+^* and *Sirt2^−/−^* mice upon IL4 treatment. Results are expressed as the mean ± SEM. *P<0.05; **P<0.01; ***P<0.001.

Considering that SIRT2 deficiency does not alter the homeostasis of the intestinal epithelium under normal conditions, we hypothesized that the contribution of the immune cell compartment may be the primary event causing more severe DSS-induced colitis in the *Sirt2^−/−^* animals. Therefore we examined the immune status of bone marrow-derived macrophages (BMDMs) from *Sirt2^+/+^* and *Sirt2^−/−^* mice. Under basal conditions, gene expression analysis in *Sirt2^−/−^* BMDMs showed an induction of the transcripts of pro-inflammatory cytokines, including *Il1β*, *Tnfα*, *Il6*, and *Mcp-1* (Figure 6A–B), and the down-regulation of anti-inflammatory genes, like the *Il4 receptor* (*Il4r*) and *Il10* (Figure 6C), highlighting a pro-inflammatory state in *Sirt2^−/−^* BMDMs in basal conditions.

In response to different stimuli, macrophages may undergo classical M1 or alternative M2 polarization. The M1 phenotype is characterized by the expression of high levels of pro-inflammatory cytokines, while M2 macrophages are considered to have immunosuppressive functions [22]. According to the observed gene expression patterns (Figure 6B–C), BMDMs from *Sirt2^+/+^* and *Sirt2^−/−^* mice possess different phenotypic profiles. We hence treated BMDMs with either LPS or IL4 (Figure 6D–E), which respectively are responsible for the induction of M1 or M2 polarization. LPS treatment induced the transcript levels of pro-inflammatory cytokine genes in both genotypes, but the increase was significantly more pronounced in the *Sirt2^−/−^* BMDMs (Figure 6D). Alternatively, the IL4-mediated M1-to-M2 switch is driven by the activation of the STAT6/GATA3 pathway [22]. IL4-stimulated *Sirt2^−/−^* BMDMs showed reduced *Gata3* induction together with a lower expression of *Arginase* 1 (*Arg1*) and *Cd11c* (Figure 6E), two well known M2 marker genes [23]. These data demonstrate that SIRT2 deficiency also directly affects macrophage polarization, mimicking a pro-inflammatory milieu.

### The absence of SIRT2 expression triggers the hyperacetylation of NF-κB in BMDMs

**Figure 7:**
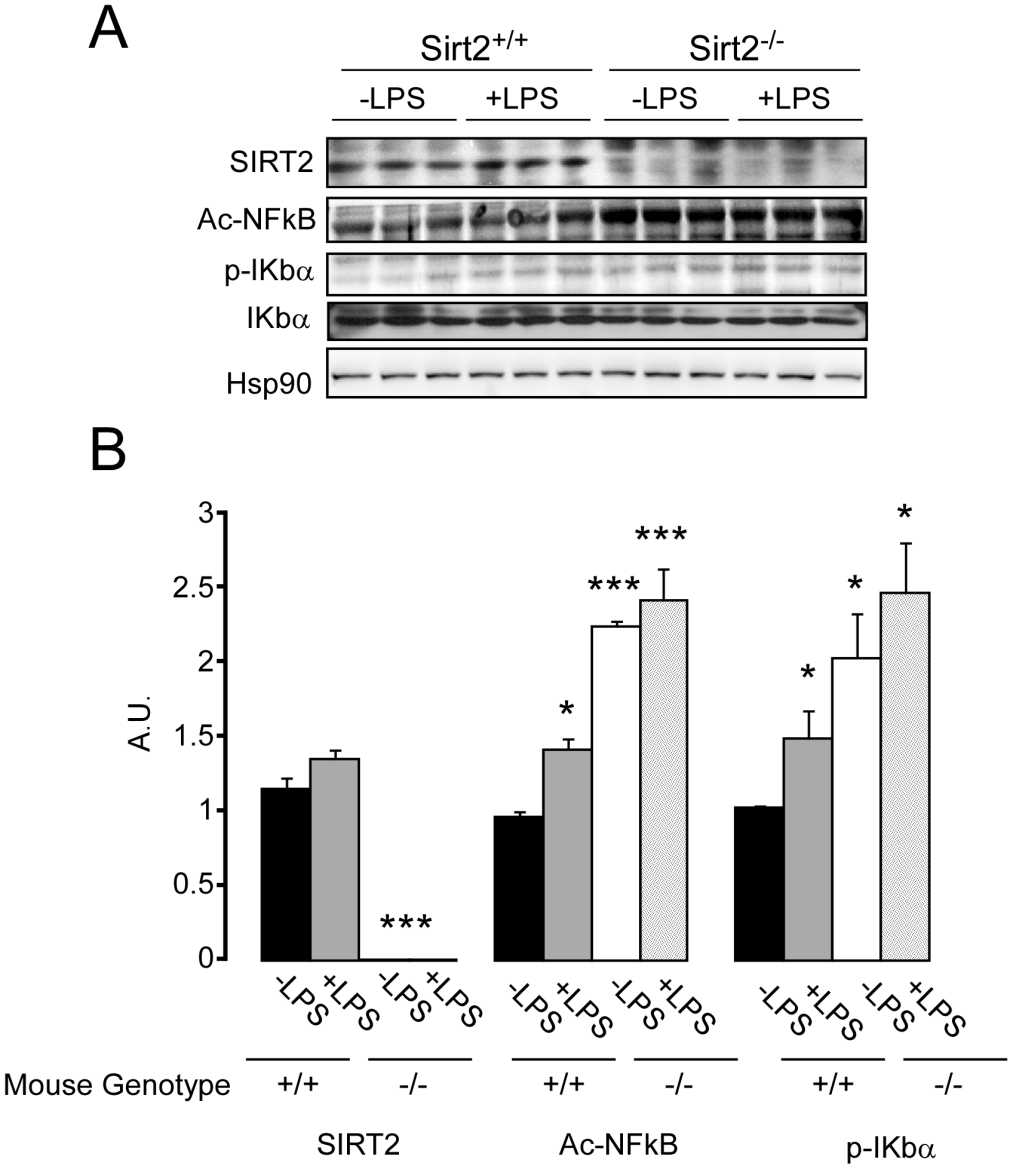
p65 subunit of NF-κB is hyperacetylated in Sirt2-deficient BMDMs. (A) Western blot analysis of the acetylated p65 subunit of NF-κB, and total- and phosphorylated-IKbα in BMDMs from *Sirt2^+/+^* and *Sirt2^−/−^* mice upon LPS treatment.(B) Quantification of the differences observed in (A) were obtained using ImageJ software. Results are expressed as the mean ± SEM. *P<0.05; **P<0.01; ***P<0.001 and the statistical significance was calculated comparing each group to untreated (−LPS) *Sirt2^+/+^*. N = 3/group.

NF-κB activation plays a fundamental role in the transcriptional regulation of inflammation-related genes and is associated with several chronic inflammatory diseases [24]. Previous findings have demonstrated a link between SIRT2 and NF-κB [4], [5], [6]. Hyperacetylation of the p65 subunit of NF-κB at Lys310, after *Sirt2* knockdown, has been linked to an increase in NF-κB-dependent transcription, causing deleterious effects on inflammatory diseases [4], [5], [6]. To gain further insight into the molecular mechanisms underlying the effects of *Sirt2* deletion on DSS-induced colitis, we investigated whether the effects observed in BMDMs were associated with alterations in the acetylation of NF-κB. *Sirt2^−/−^*BMDMs exhibited higher NF-κB acetylation under both basal and LPS-treated conditions, while a slight increase in acetylation was observed in *Sirt2^+/+^* BMDMs after LPS stimulation (Figure 7). Moreover, the basal phospho-IκBα levels in Sirt2*^−/−^* BMDMs were similar to those of LPS-treated *Sirt2^+/+^* BMDMs, confirming the constitutive activation of NF-κB in Sirt2*^−/−^* BMDMs. Our data therefore supports that SIRT2 targets Lys310 on the p65 subunit of NF-κB in BMDMs, resulting in pro-inflammatory gene expression.

## Discussion

In the present study, we identified a novel role for SIRT2 as a potential suppressor of DSS-induced colitis in the mouse [13]. First by interfering with intestinal barrier function, then stimulating local inflammation and dysplasia, DSS-induced colitis resembles the clinical progression of human UC, representing an important model for the translation of mouse data to human disease relevance [25]. Here we showed that SIRT2 deficiency led to a more severe colitis compared to that seen in *Sirt2^+/+^* control mice. Although *Sirt2^−/−^* mice were indistinguishable from *Sirt2^+/+^* mice, with respect to body weight and intestinal morphology (Figures 1 and 2), upon a DSS challenge they developed more severe colitis (Figures 3–5). Several reasons can be invoked to explain the absence of a difference in the intestinal epithelium between *Sirt2^+/+^* and *Sirt2^−/−^* mice under basal conditions. First, *Sirt2* belongs to the family of Sirtuins, with 7 different members (*Sirt1-7*) that are evolutionary conserved. The loss-of-function of one of the Sirtuins could hence cause a compensatory increase in the function of other family members. Second, intestinal epithelium undergoes a very fast proliferation/differentiation/death cycle, due to the necessity for continuous cell renewal. This fast regeneration of intestinal cells implies the presence of several redundant checkpoints and makes it unlikely that the mutation of a single gene, unless it is a master regulator of proliferation (like *β-catenin*, *p53*, *Notch*, etc), exerts a dominant effect under basal conditions.

However upon DSS challenge, together with increased acceleration in body weight loss and rectal bleeding, intestinal permeability and histological score were exacerbated. Concordantly, a more detailed histological analysis of the intestine showed an increase in the extent of erosion/ulceration, and leukocytic infiltration density with mural involvement (Figure 3). The increased inflammation in *Sirt2^−/−^* mice was also highlighted by the measurements of cytokine levels in the plasma and cytokine mRNA expression in colon tissue. TNFα and IL1β levels in the plasma and their transcript levels in the colon were increased in *Sirt2^−/−^* mice following DSS, while basal upregulation was limited and only observed in colon tissue mRNA measurements (Figure 5). In addition, the increased intestinal permeability in *Sirt2^−/−^* mice upon DSS treatment was underscored by their more pronounced reduction of colon TJ proteins, *Ocln*, *Cldn1*, and *Zo1*, when compared to *Sirt2^+/+^* animals.

Although germline *Sirt2^−/−^* mice represent a reliable genetic model to study the pathophysiology of IBD, it is difficult to determine the potential involvement of different tissues, including intestine and immune cell compartment, in the development of colitis. However, both morphological and gene expression analysis in basal conditions defined the role of the intestinal tissue in the development of colitis as secondary (Figure 2 and 5). Since the inflammatory cells of the gut are initially recruited from the bone marrow compartment [26], bone marrow derived macrophages (BMDMs) from *Sirt2^+/+^* and *Sirt2^−/−^* mice were isolated and examined *ex vivo* for functional differences. Notably, macrophages have the unique ability to respond to environmental cues by taking on one of two functional phenotypes designated as pro-inflammatory M1 and anti-inflammatory M2 macrophages. Classically activated M1 cells are implicated with initiating and sustaining inflammation, while M2 cells are associated with the resolution of chronic inflammation [27]. These two distinct phenotypes, M1 and M2, can be induced *ex vivo* by treating BMDMs with LPS or IL4, respectively. Before macrophage activation, we found that *Sirt2* deficiency resulted in the basal induction in the transcript levels of pro-inflammatory cytokine genes in BMDMs (Figure 6). Moreover, upon LPS stimulation and M1 phenotype induction, differences observed in *Sirt2^−/−^* mice-derived BMDMs under basal conditions became accentuated (Figure 6). In addition, *Sirt2^−/−^* BMDMs exhibited reduced basal mRNA expression levels for the M2-associated anti-inflammatory cytokine *Il10* and the *Il4 receptor* [28], [29]. IL4R activates the STAT6/GATA3 signaling cascade, which in turn controls transcription of genes typical of M2 polarization (e.g. *Arg1*, *Cd11c*) [30]. Correspondingly, after IL4 stimulation, *Gata3* [22], *Arg1*, and *CD11c* [23] mRNA expression is reduced in *Sirt2^−/−^* mice. In addition, the hyperacetylation of the p65 subunit of NF-κB in *Sirt2^−/−^* BMDMs may mechanistically contribute to the pro-inflammatory effect exerted by *Sirt2* deficiency. Consistent with previous studies [4], [5], hyperacetylation of NF-κB induces its activity, triggering inflammatory pathways. Moreover, it has been previously demonstrated that the IL4/STAT6/GATA3 pathway negatively regulates NF-κB-dependent gene expression [31]. Thus, the inhibition of the IL4-dependent activation may unhinge an important repressive mechanism used to counterbalance the inflammatory process. These data hence demonstrate that *Sirt2* deficiency predisposes to and promotes inflammation, while also inactivating the classical M2-associated anti-inflammatory pathways, as a primary cause for the more severe development of colitis in these animals.

Our results demonstrate a potential role for SIRT2 as a suppressor of colitis in the mouse. This also leads us to speculate that the activation of SIRT2 may be a potential approach to treat inflammatory bowel disease. Further investigations to identify therapies targeting SIRT2 to improve inflammatory bowel disease are hence warranted. Moreover, these findings also highlight the novel role of SIRT2 in macrophage M1/M2 polarization, which could have far reaching implications for other inflammatory- diseases.
